# Transcriptome analysis of adipocytokines and their-related LncRNAs in lung adenocarcinoma revealing the association with prognosis, immune infiltration, and metabolic characteristics

**DOI:** 10.1080/21623945.2022.2064956

**Published:** 2022-04-17

**Authors:** Jie Ren, Hui Zhang, Jinna Wang, Yingsong Xu, Lei Zhao, Qihang Yuan

**Affiliations:** a Department of Oncology, First Affiliated Hospital of Dalian Medical University, Dalian, Liaoning, China; b Clinical Laboratory of Integrative Medicine, First Affiliated Hospital of Dalian Medical University, Dalian, Liaoning, China; c Department of General Surgery, First Affiliated Hospital of Dalian Medical University, Dalian, Liaoning, China; d Department of Oncology, Dalian Friendship Hospital Affiliated to Dalian Medical University, Dalian, Liaoning, China; e Department of Thoracic Surgery, First Affiliated Hospital of Dalian Medical University, Dalian, Liaoning, China

**Keywords:** Lung adenocarcinoma, adipocytokine, long non-coding RNA, prognostic signature, immune infiltration

## Abstract

Lung adenocarcinoma (LUAD) is amongst the major contributors to cancer-related deaths on a global scale. Adipocytokines and long non-coding RNAs (lncRNAs) are indispensable participants in cancer. We performed a pan-cancer analysis of the mRNA expression, single nucleotide variation, copy number variation, and prognostic value of adipocytokines. LUAD samples were obtained from the Gene Expression Omnibus (GEO) and The Cancer Genome Atlas (TCGA) databases. Simultaneously, train, internal and external cohorts were grouped. After a stepwise screening of optimized genes through least absolute shrinkage and selection operator regression analysis, random forest algorithm,, and Cox regression analysis, an adipocytokine-related prognostic signature (ARPS) with superior performance compared with four additional well-established signatures for survival prediction was constructed. After determination of risk levels, the discrepancy of immune microenvironment, immune checkpoint gene expression, immune subtypes, and immune response in low- and high-risk cohorts were explored through multiple bioinformatics methods. Abnormal pathways underlying high- and low-risk subgroups were identified through gene set enrichment analysis (GSEA). Immune-and metabolism-related pathways that were correlated with risk score were selected through single sample GSEA. Finally, a nomogram with satisfied predictive survival probability was plotted. In summary, this study offers meaningful information for clinical treatment and scientific investigation.

## 1. Introduction

Throughout the globe, lung cancer has been acknowledged to be among the major contributor to cancer-related fatalities. Lung adenocarcinoma (LUAD) is currently the most prominent kind of lung cancer, accounting for about 50% of all cases with respect to histologic type and prognosis. The incidence of LUAD is growing every year, particularly in women and young people. It is estimated that the five-year rate of survival for LUAD patients is less than 20%, despite advancements in diagnosis and therapy [1,2]. The lack of comprehensive comprehension of the underlying mechanisms of LUAD makes it difficult to enhance the treatment benefits. As a consequence, it is necessary to establish a unique prognostic signature that would allow for more accurate anticipation of LUAD prognosis.

Adipocytokines, secreted by adipocytes, play a function to communicate vital organs in order to sustain metabolic homoeostasis while also possessing the ability to regulate the inflammatory response [3]. As a consequence, the dysfunction of adipocytokines has been shown to be a contributing factor in a broad variety of illnesses. Interestingly, the understanding of the infiltration of macrophages into the adipose tissue not only offered an insight into the source of adipose-derived cytokines but also illustrated the close juxtaposition between metabolic and immune cells in metabolic organs for the first time [4,5]. What’s more, either the relationship between adipose tissue dysfunction and cancer cachexia [6] or complicated signalling pathways that underlie the obesity-cancer link [7] imply the potential role of adipocytokines in cancer. In addition, long non-coding RNAs (lncRNAs) have recently been shown to have the ability to modulate the expression of genes at several levels, such as post-transcriptional, transcriptional, and epigenetic [8], and their role in the occurrence and progression of cancer is very crucial [9].

Hence, the aim of the present research was to explore the potential function of adipocytokines in pan-cancer so as to construct a new adipocytokine-related prognostic signature (ARPS) to distinguish low- and high-risk LUAD patients and illustrate the potential discrepancies of immune and metabolism features in patients with different prognosis. Last but not least, a nomogram was developed for anticipating the survival rates of LUAD patients which can be used to support clinical decision-making as well as individual management. We are sure that the findings of the present research shed new light on the diagnosis and treatment of LUAD and provide a theoretical foundation for more thorough researches in the future.

## 2. Materials and methods

### 2.1. Data collection

The National Human Genome Institute and the National Cancer and Cancer Institute debuted the Cancer Genome Atlas (TCGA) system in 2006 with the ambition of mapping cancer genes, thereby understanding the underlying pathways of cancer, and improving the capacity to inhibit cancer progression, make an accurate diagnosis, and cure cancer. The Gene Expression Omnibus (GEO) is an international open-source repository for high-throughput microarray and next-generation sequence gene function data sets that have been reported by the academic community. In the present research, mRNA expression profiles, single nucleotide variation (SNV), copy number variation (CNV), and corresponding clinical characteristics of pan-cancer transcriptomes were obtained from the TCGA database. The GEO database was also employed to acquire the transcriptome profiles as well as the clinical features of LUAD patients. ‘KEGG_ADIPOCYTOKINE_SIGNALING_PATHWAY’ gene set containing 67 adipocytokines and ‘c2.cp.kegg.v7.4.symbols.gmt’ file were obtained from the Molecular Signatures Database (MSigDB) [10–12]. The Ensembl database provided the human gene transfer format (gtf) file that was used in the present research [13].

### 2.2. Data procession

The intersection of transcriptome profiles from TCGA and transcriptome profiles from GEO were taken to obtain intersecting genes. Expression data of intersecting genes from the TCGA dataset and GEO dataset were converted into log2(x + 1) form and batch normalized by utilizing the ‘sva’ package in R. LncRNAs were separated from protein-coding genes according to the gtf file utilizing Perl languages. The transcriptome data of involved adipocytokines and lncRNAs were collected respectively. The Pearson correlation coefficients between lncRNAs and the adipocytokines were computed by the built-in function ‘cor. test’ in R. Adipocytokine-related lncRNAs were selected with P values < 0.001 and |correlation coefficients| > 0.4. The transcriptome data of the adipocytokines and their-related lncRNAs were integrated with corresponding clinical data in TCGA and GEO datasets.

### 2.3. Pan-cancer analysis

In recent years, many research studies have been carried out on the relationship investigation between adipocytokines and cancers. However, the variations of adipocytokines in a variety of cancers are not well summarized. For a pan-cancer overview about variations of adipocytokines, SNV and CNV data derived from the TCGA database were analysed and visualized in the form of heat maps. Additionally, pan-cancer evaluation of differential mRNA expression was conducted. What’s more, univariate Cox regression analysis was conducted to identify the prognostic significance of adipocytokines in various cancers. All these analyses were conducted by R and TBtools[14].

### 2.4. Construction, validation, and comparative analysis of the ARPS in LUAD

In the following section, we focused on LUAD for a deep and comprehensive study. First and foremost, LUAD samples with complete transcriptome data and survival time in the TCGA dataset were classified at random at a ratio of 1:1 into a training cohort and test1 cohort. Subsequently, all samples from the TCGA dataset were assigned to the test2 cohort, whereas all samples from the GEO cohort were assigned to test3.

In the training cohort, univariate Cox regression analysis was conducted to search for genes with prognostic value utilizing coxph function of ‘survival’ in R (filter criteria: p < 0.05). Then, the R software’s ‘randomForestSRC’ and ‘randomSurvivalForest’ packages were employed to further assess the importance of these genes to prognosis outcomes [15]. Afterwards, genes with raw importance>0 and relative importance>0.38 were selected for least absolute shrinkage and selection operator (LASSO) regression analysis to eliminate collinearity and prevent over-fitting. After obtaining the most appropriate variables, multivariate Cox regression analysis was utilized to create an adipocytokine-related prognostic signature (ARPS) and the risk score of each sample was derived utilizing the equation below: risk score = $\overset{n}{\underset{k=1}{\sum }}expk*\beta k$. Following the computation of the risk score, the samples in the training cohort were divided into two subgroups depending on the median risk score: low- and high-risk subgroups. In a manner consistent with the median risk score achieved in the training cohort, all of the samples in the test1, test2, and test3 cohorts were divided into low- and high-risk, for further analysis.

The subsequent analyses were performed on the training, test1, test2, and test3 cohorts for the purpose of external and internal validation of ARPS: (1) the use of principal component analysis (PCA) to visualize sample classification; (2) using the ‘pheatmap’ R package to create a heatmap depicting the expression levels of the genes implicated in ARPS; (3) The Kaplan-Meier approach was used for the purpose of performing a survival analysis to determine if the signature has the potential to predict survival; (4) We established multivariate receiver-operating characteristic (multi-ROC) curves for comparing the diagnostic value of the risk score with other clinical prognostic features, such as stage, smoking history, gender, and age based on the area under the curve (AUC); (5) To elaborate the association between ARPS and clinicopathological traits, the ‘fisher.test’ function in R was implemented to investigate the discrepancy in the distributions of survival status, age, gender, smoking history, and tumour stage between the low-risk and high-risk populations.

The prediction accuracy was evaluated by comparing our ARPS with four additional prognostic signatures (an immune-related signature created by Dina Guo et al [16], an autophagy-related gene prognostic signature created by Jie Zhu et al [17], and a seven-gene signature created by Yingqing Zhang et al [18], and a glycolysis-related signature created by Lei Zhang et al [19]) as follows: Genes used for constructing these signatures were obtained; transcriptome data and survival time were prepared; the R software’s ‘survival’, ‘tidyverse’, and ‘timeROC’ packages were utilized.

### 2.5. The discrepancy of the tumour microenvironment (TME), immune checkpoint genes (ICGs) expression, immune subtypes, and immune response in low- and high-risk cohorts

In view of the association between adipocytokines and immune, whether immune-related discrepancies can be distinguished based on ARPS and whether there is a corresponding association between immune-related discrepancies and prognosis were explored as follows. First, tumour microenvironment (TME) was paid attention to and the ESTIMATE algorithm was utilized to compute the Immunescore, StromalScore, ESTIMATEScore, and TumorPurity for each sample based on transcriptome data using the ‘estimate’ package in R. Notably, an elevation in the score was correlated with an increase in the percentage of the matching TME components. Afterwards, the differential expression analysis of 47 common ICGs in high- and low-subgroups were conducted and only the statistically significant results were shown (filter criteria: p < 0.05). Then an approach, published in the ‘Immunity’ journal in 2018 [20], was utilized to identify the immune subtype of each sample in train, test 1, and test 2 cohorts respectively. There were six immune subtypes in total (i.e. wound healing (C1), IFN-γ dominant (C2), inflammatory (C3), lymphocyte depleted (C4), immunologically quiet (C5), and TGF-b dominant (C6)). The composition discrepancy of immune subtypes in various subgroups was analysed utilizing the chi-square test. For in-depth investigation of immune components in the TME, the EPIC, QUANTISEQ, CIBERSORT-ABS, MCPCOUNTER, TIMER, XCELL, and CIBERSORT algorithms were utilized to evaluate immune responses between low- and high-risk subgroups on the basis of ARPS.

### 2.6. ARPS-based functional annotation

Numerous reliable sources have been developed during the last 3 decades to gather and organize reactomes and pathways on the basis of standard biochemical understanding, such as the Kyoto Encyclopaedia of Genes and Genomes (KEGG) [21], which provided functional annotations in various cancers. To identify abnormal pathways underlying the low-and high-risk subgroups, gene set enrichment analysis (GSEA) was utilized for KEGG analysis that was performed with the aid of GSEA (version: 4.1.0) [10,11]. In view of the association between adipocytokine and immune and metabolism [22–25], 43 metabolism-related pathways and 33 immune-related pathways were distinguished respectively based on the ‘c2.cp.kegg.v7.4.symbols.gmt’ file from the MSigDB [12]. The activities of these pathways were evaluated utilizing a single sample gene set enrichment analysis (ssGSEA) in R according to the transcriptome data of individual samples. Subsequently, utilizing the built-in function ‘cor. test’ in R, the Pearson correlation coefficient between risk score and the activities of metabolism-related and immune-related pathways were computed.

### 2.7. Construction and validation of a nomogram

First and foremost, univariate and multivariable Cox regression analyses were also respectively carried out in order to determine whether the risk score had prognostic significance independently. The markers that showed statistical significance (p < 0.05) in both the univariate and multivariate Cox regression analyses in training, test1, test2, and test3 cohorts were deemed to be independent prognostic variables in the sample population. Following this, a nomogram was created by integrating the independent prognostic factors with satisfied diagnostic values utilizing the ‘rms’ package in R, and subsequent calibration curves for survival probability over one, three, and five years were charted to determine the extent of fitting between the survival rates estimated by the nomogram and the actual rates.

## 3. Results

### 3.1. Data procession

**Figure 1.**
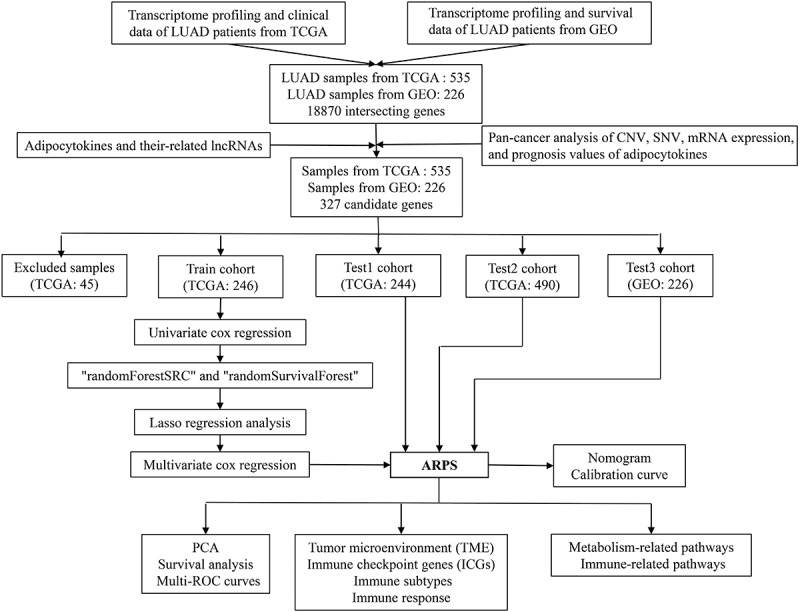
The workflow of the current study.

An overview of the research steps is presented as a flow chart in Figure 1. mRNA expression profiles, SNV, CNV, and survival data of 67 adipocytokines in all types of cancers were acquired from TCGA for pan-cancer analysis. 535 LUAD samples from the TCGA database, as well as an additional 226 LUAD samples (GSE31210) from the GEO database, were included for specific analyses in LUAD. Following the intersection of all the genes from the 2 LUAD datasets, a total of 18,870 shared genes were discovered. Of note, the GSE31210 dataset lacks the expression of the CPT1B gene; thus, 66 adipocytokines left and 261 adipocytokine-related lncRNAs with complete mRNA expression data in all samples were obtained. Then 45 LUAD samples whose survival data was not complete from the TCGA database were excluded. The remaining 490 LUAD samples obtained from the TCGA dataset together with 226 LUAD samples obtained from the GEO dataset were accompanied by complete mRNA expression data and survival data.

### 3.2. Pan-cancer introduction about variations of adipocytokines

**Figure 2.**
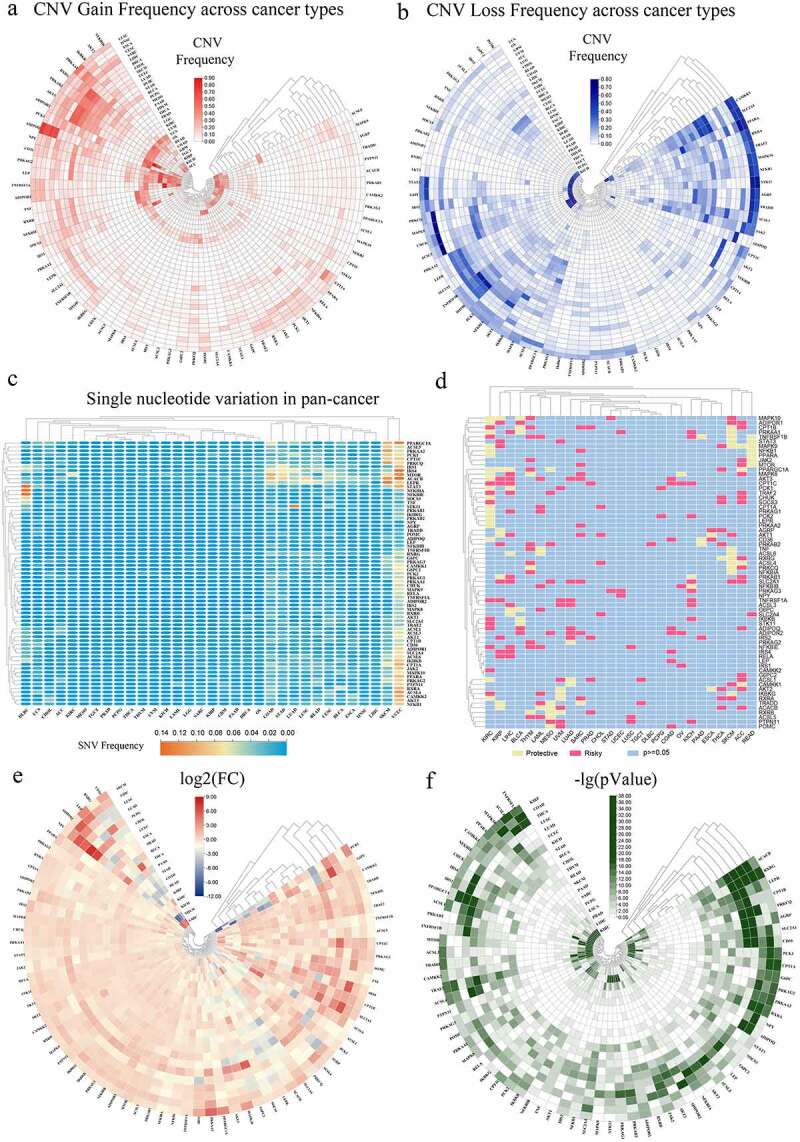
Panoramic view of adipocytokines in pan-cancer. (a) The gain frequencies of copy number variation (CNV) in diverse types of cancers. (b) Loss frequencies of CNV in diverse types of cancers. (c) Single nucleotide variation (SNV) in pan-cancer. (d) Survival landscape of adipocytokines across cancer types. (e) The changes of mRNA expression of adipocytokines across cancer types (FC: Fold changes). (f) The relevant -logP value of the changes of each gene across various cancers.

To summarize and visualize the variation of adipocytokines in various cancers, CNV and SNV data were visualized as a heat map. Figure 2(a) depicts the CNV gain frequency heatmap demonstrating that adipocytokines exhibit elevated gain frequencies in LUSC, HNSC, ESCA, CESC, SARC, USC, OV, KICH, and ACC. RXRG, PRKAB2, AKT3, ADIPOQ1, NPY, CD36, PRKAG2, and LEP obtained gain variations in a vast majority of cancers. Furthermore, Figure 2(b) depicts the CNV loss frequency heatmap, where adipocytokines had higher frequencies of loss variations in UCS and OV. The loss variations of CAMKK1, SLC2A4, ACSL1, and JAK2 existed in almost all cancers. What’s more, the heat map generated from the SNV data in Figure 2(c) revealed that a wide range of adipocytokines had SNV in UCEC and the SNV of ACACB was involved in multiple cancers.

### 3.3. Pan-cancer analysis of the prognostic value and mRNA expression of adipocytokines

According to contemporary cancer research, aberrant mRNA expression might indicate that the relevant gene has a high likelihood to perform a critical function in disease progression [26–28]. Then the univariate cox regression analysis between the mRNA expression and overall survival (OS) was conducted to identify risky adipocytokines with HR>1 and p < 0.05 and protective adipocytokines with HR<1 and p < 0.05 (Figure 2(d)). For a visual exhibition, mRNA expression levels of adipocytokines were depicted in Figure 2(e). In the heat map, NPY showed an obviously increasing expression in many cancer types, such as SARC, THCA, CHOL, PCPG, LUAD, LUSC, and LIHC. ADIPOQ, LEP, RXRG, and CD36 had simultaneous low expression in THYM and READ, while simultaneously highly expressed in SARC. Moreover, simultaneous down-regulation of PCK1 and G6PC existed in SARC, KICH, KIRC, KIRP, COAD, STAD, and an opposite trend emerged in LUAD. All these conclusions were based on |log2(FC)| >2. To more clearly show the salience of the variation in the levels of mRNA expression, a heat map of the corresponding -lg (pValue) was created. The greener the colour, the more violent the alteration in mRNA expression in the relevant malignancy (Figure 2(f)).

### 3.4. Construction and comparative analysis of the ARPS in LUAD

In the following specific analyses about LUAD, 490 LUAD samples in the TCGA dataset, as well as 226 LUAD samples in the GEO dataset with complete mRNA expression data and survival data, were included for group division. In total, there are four cohorts in this study: training cohort (246 TCGA samples), test1 cohort (244 TCGA samples), test2 cohort (all of the 490 TCGA samples), and test3 cohort (226 GEO samples). Notably, the test 1, as well as test 2 cohorts, were utilized for internal validation, whereas the test3 cohort was utilized for external validation during the course of the ARPS validation.

**Figure 3.**
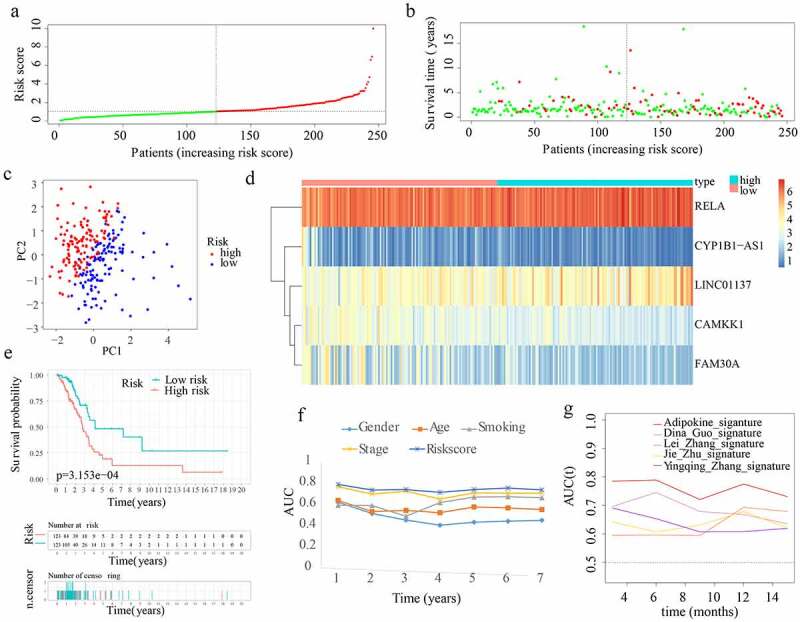
Construction of ARPS in the training cohort. (a) Group division according to median risk score in the training cohort. (b) Survival status and risk score distributions in the training cohort. (c) Training cohort’s PCA. (d) In the training cohort, a heatmap depicting the levels of expression of the 5 genes associated with the signature. (e) Survival curve of the training cohort. (f) AUC values of multi-ROC curves in the training cohort. (g) The AUC values of ROC curves for prediction ability of ARPS in comparison to four additional signatures in the training cohort.

In total, 66 adipocytokines and 261 adipocytokine-related lncRNAs were merged together as candidate genes for signature construction. In the training cohort, 53 adipocytokines and their-related lncRNAs with prognostic values were selected from the 327 candidate genes after conducting univariate Cox regression analysis. After assessing the importance of the 53 genes to prognosis outcome, 24 crucial candidate genes were identified through random forest analysis. Supplementary Figure S1(a) depicts the correlation between the error rate and the number of classification trees, as well as the order of the 24 critical genes’ out-of-bag relevance. Following this, LASSO regression analysis was conducted to remove collinearity among the 24 crucial genes and prevent the prognostic model from over-fitting. Then 12 genes were obtained for subsequent multivariate Cox regression analysis (Supplementary Figure S1(b,c)). Eventually, a multivariate Cox proportional hazards regression analysis was performed by incorporating two adipocytokines and three adipocytokine-related lncRNAs (i.e. CYP1B1-AS1, RELA, CAMKK1, FAM30A, and LINC01137) to create an innovative ARPS. Based on the Cox coefficient obtained through multivariate Cox regression analysis, the following equation was employed to determine the risk score: (0.811434858341342 * RELA expression level) – (0.663459367611146 * CAMKK1 expression level) + (0.380891381331339 * LINC01137 expression level) – (0.366850416554157 * FAM30A expression level) – (0.479894025470768 * CYP1B1-AS1 expression level). Subsequently, using the value 1.028679688 as the median risk score, samples from the training cohort were split into low- and high-risk subgroups (Figure 3(a)). The survival status and risk score distributions suggested that patients having higher risk scores had a greater likelihood of death (Figure 3(b)). The PCA findings depicted in Figure 3(c) reveal that patients belonging to the two subgroups might be readily discriminated against. Using a heatmap as shown in Figure 3(d), we can visualize the levels of expression of the 5 genes that are implicated in this model, which were also closely aligned with the coefficients in the aforementioned equation. The genes LINC01137 and RELA were discovered to be more strongly expressed in the high-risk subgroup as opposed to the low-risk subgroup. On the other hand, the genes CYP1B1-AS1, CAMKK1, and FAM30A were found to be expressed at lower levels in the high-risk subgroup. According to Figure 3(e), a lower OS rate was constantly observed among individuals in the high-risk subgroup (p < 0.05). Furthermore, the AUC values with regard to the survival probability of the ROC curves of risk score were 0.790, 0.737, 0.741, 0.715, 0.741, 0.756, and 0.738 for one, two, three, four, five, six, and seven years (Figure 3(f)), which were higher than other clinical indicators and implicitly indicates that ARPS may exert a strong prediction role of survival for LUAD patients. To elaborate the association between ARPS and clinical traits of the samples chosen to performed the analysis above, the discrepancy in the distributions of survival status, age, gender, smoking history, and stage between the low-risk and high-risk population in train cohort were displayed in Supplementary Figure S2(a). Of note, more stage I samples existed in low-risk subgroup. Furthermore, our ARPS had an obviously superior probability to predict survival compared to four additional prognostic signatures (Figure 3(g)).

### 3.5. External and internal verification of the ARPS in LUAD

**Figure 4.**
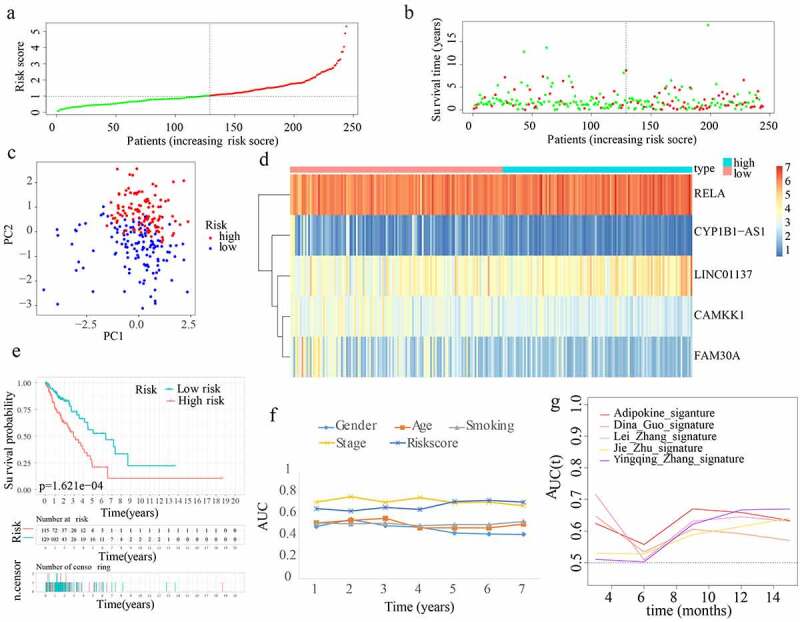
Internal verification of ARPS in test1 cohort. (a) In the test 1 cohort, there was a classification into subgroups. (b) Survival status and risk score distributions of test 1 cohort. (c) PCA of test 1 cohort. (d) The heatmap depicting the levels of expression of the 5 genes implicated in the signature in test1 cohort. (e) Survival curve of test 1 cohort. (f) AUC values of multi-ROC curves in test 1 cohort. (g) The AUC values of ROC curves for prediction ability of ARPS in comparison to four additional signatures in test1 cohort.

**Figure 5.**
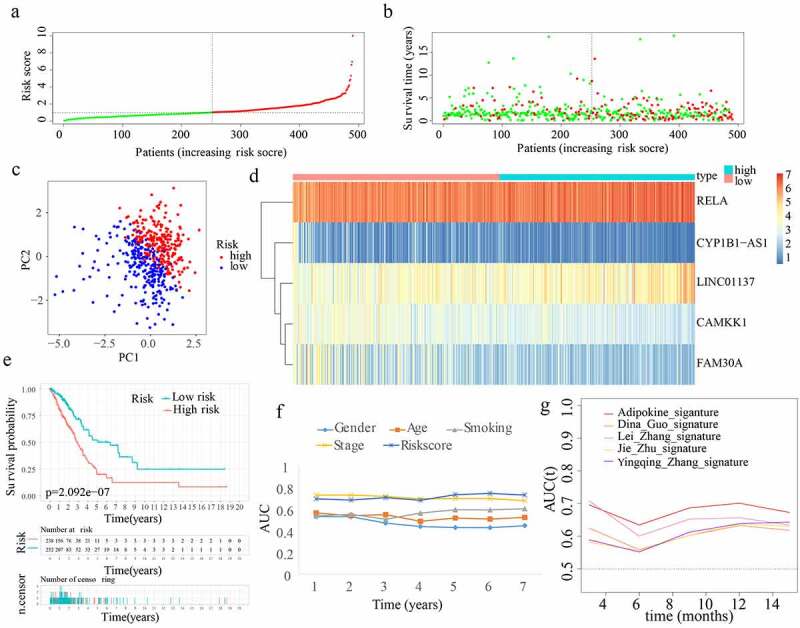
Internal verification of ARPS in test 2 cohort. (a) Classification of test 2 cohort into subgroups. (b) Survival status and risk score distributions of test 2 cohort. (c) PCA of test2 cohort. (d) Heatmap depicting the levels of expression of the 5 genes related to the signature in test2 cohort. (e) Survival curve of test 2 cohort. (f) AUC values of multi-ROC curves in test 2 cohort. (g) The AUC values of ROC curves for ARPS prediction ability in comparison to four additional signatures in test 2 cohort.

**Figure 6.**
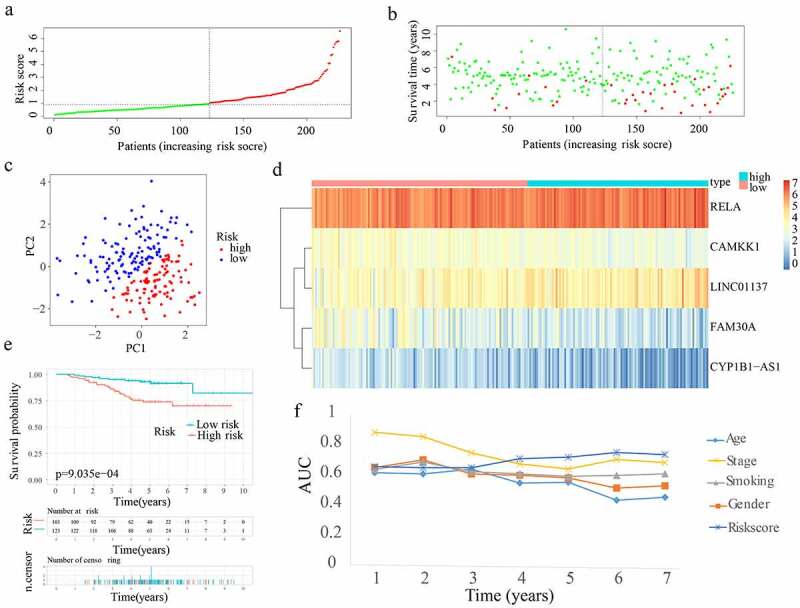
External verification of ARPS in test 3 cohort. (a) Test 3 cohort was divided into groups. (b) Survival status and risk score distributions of test 3 cohort. (c) PCA of test3 cohort. (d) Heatmap illustrating the levels of expression for the five genes linked to the signature in test 3 cohort. (e) Survival curve of test3 cohort. (f) AUC values of multi-ROC curves in test 3 cohort.

Risk scores were generated and samples were divided in the 3 cohorts for the purpose of evaluating the validity and reliability of the ARPS: test1, test2, and test3 cohorts (Figure 4(a), 5(a), 6(a)). Notably, the median risk score of 1.028679688 found in the training cohort served as a unified benchmark for separating the samples. For both the internal validation (test 1 and test2 cohorts) as well as the external validation (test3 cohort), the survival status and risk scores distributions displayed comparable trends to the ones found in the training cohort (Figure 4(b), 5(b), 6(b)). By means of PCA, it was proved that patients in the two subgroups might be readily discriminated from one another (Figure 4(c), 5(c), 6(c)). The heatmaps generated from the test1, test2, and test3 cohorts revealed the presence of genes (LINC01137 and RELA) with high expression and genes (CYP1B1-AS1, CAMKK1, and FAM30A) with attenuated expression in the high-risk subgroup (Figure 4(d), 5(d), 6(d)). Moreover, in the test1, test2, and test3 cohorts, individuals with high-risk scores reported unfavourable OS rates (all p < 0.05) (Figure 4(e), 5(e), 6(e)). Additionally, the multi-index ROC curves were created for test1, test2, and test3 cohorts. The ROC curve AUC values of different clinical indicators suggested that the diagnostic value of risk score is as good as tumour stage and obviously better than age, gender, and smoking history (Figure 4(f), 5(f), 6(f)). In terms of clinical data difference between low-risk and high-risk populations, more late-stage samples occurred in the high-risk subgroup whereas more early-stage samples existed in the low-risk subgroup in test1, test2, and test3 cohorts (Supplementary Figure S2(b–d)). Similarly, in test 1 and test2 cohorts, our ARPS also showed visible advantages of the probability to predict survival compared to four additional prognostic signatures (Figure 4(g), 5(g)).

### 3.6. The discrepancies of the immune microenvironment (TME), immune checkpoint genes (ICGs) expression, immune subtypes, and immune response in low- and high-risk subgroups

**Figure 7.**
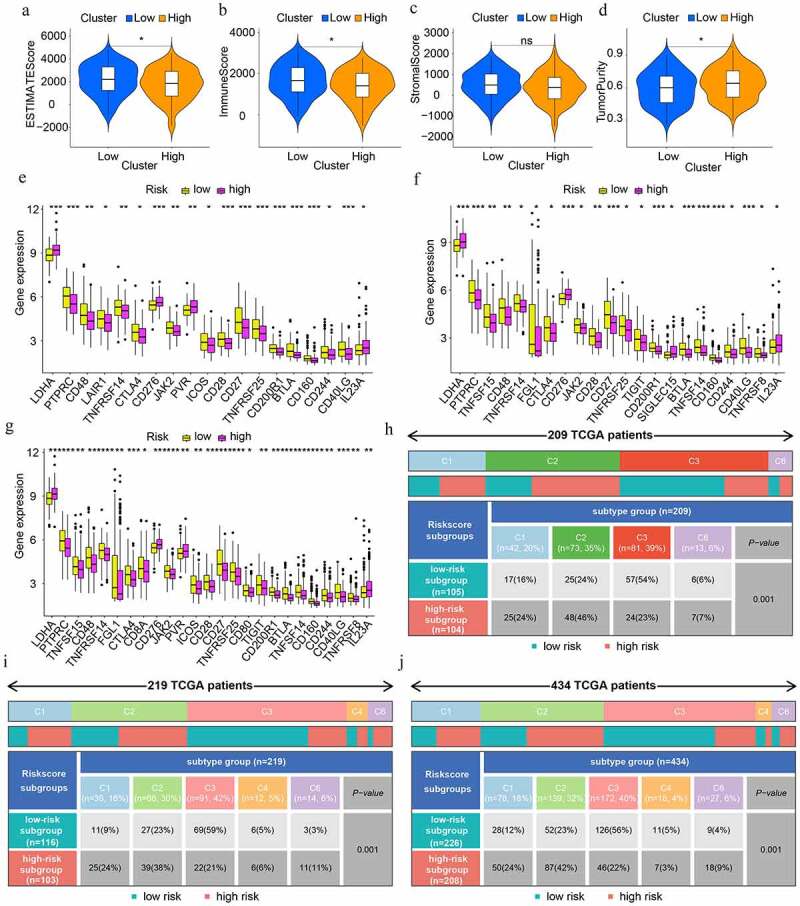
The discrepancies in tumour microenvironment (TME), immune checkpoint genes (ICGs), and immune subtypes in low- and high-risk population. (a-d) TME scores in high- and low-risk subgroups. Greater scores denote an elevated proportion of the matching TME component. (e-g) Differential expression analysis of ICGs in train, test1, and test2 cohorts respectively. (h-j) Heat map and table showing the distribution of immune subtypes (C1, C2, C3, C4, C5, and C6) between the risk score-based subgroups in train, test1, and test2 cohorts respectively.

In order to explore whether the change of TME is associated with the prognosis in LUAD, a rudimentary understanding of the discrepancy of TME was shown in Figure 7(a–d). In the training cohort, lower ESTIMATEScore and ImmuneScore which are in agreement with higher tumor purity in the high-risk subgroup imply an immunosuppressive environment in the high-risk subgroup. With an impact on TME, the different expressions of ICGs were compared subsequently. Compared with the low-risk subgroup, LDHA, CD276, and IL23A expressed highly while PTPRC, CD48, TNFRSF14, CTLA4, JAK2, CD28, CD27, TNFRSF25, CD200R1, BTLA, CD160, CD244, and CD40LG had lower levels of expression in the high-risk subgroup in training, test1, and test2 cohorts (Figure 7(e–g)). For an intensive investigation of TME, the discrepancy of immune subtypes in the three cohorts were identified respectively. It inferred that there were more samples of the C3 subtype in the low-risk subgroup, while more samples of C1 and C2 subtypes in the high-risk subgroup (p < 0.001) (Figure 7(h–j)). Notably, more SNV neoantigens, higher intratumor heterogeneity, stronger proliferation, more Th2 cells, and few Th17 cells existed in C1 and C2 compared with C3 subtypes [20].

**Figure 8.**
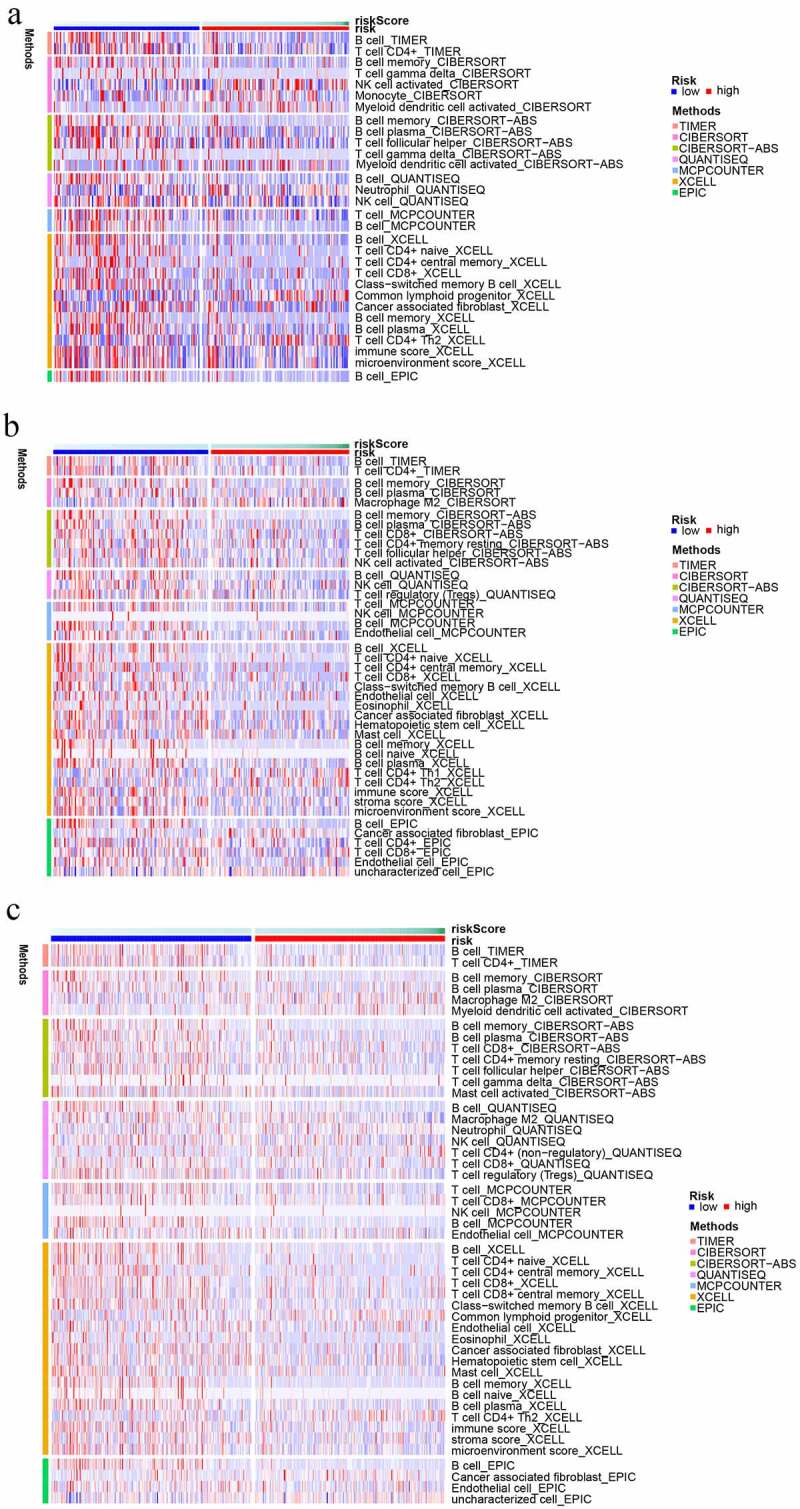
The immune cell infiltration distribution landscape in (a) train, (b) test 1, and (c) test 2 cohorts.

In addition, the immune responses were explored by means of EPIC, MCPCOUNTER, QUANTISEQ, CIBERSORT-ABS, XCELL, TIMER, and CIBERSORT algorithms and were shown in visual heat maps. After synthesizing all the differences between low- and high-risk subgroups in train, test1, and test2 cohorts, we discovered that a lower proportion of various types of B cells existed in the high-risk subgroup. In addition, nearly all types of T cells illustrated an elevated proportion in the high-risk subgroup including follicular helper T cell (Tfh). Notably, T helper 2 (Th2) cells have an elevated proportion in the high-risk subgroup and T cell regulatory (Treg) cells have an elevated proportion in low-risk subgroups (Figure 8(a–c)).

### 3.7. ARPS-based functional annotation

**Figure 9.**
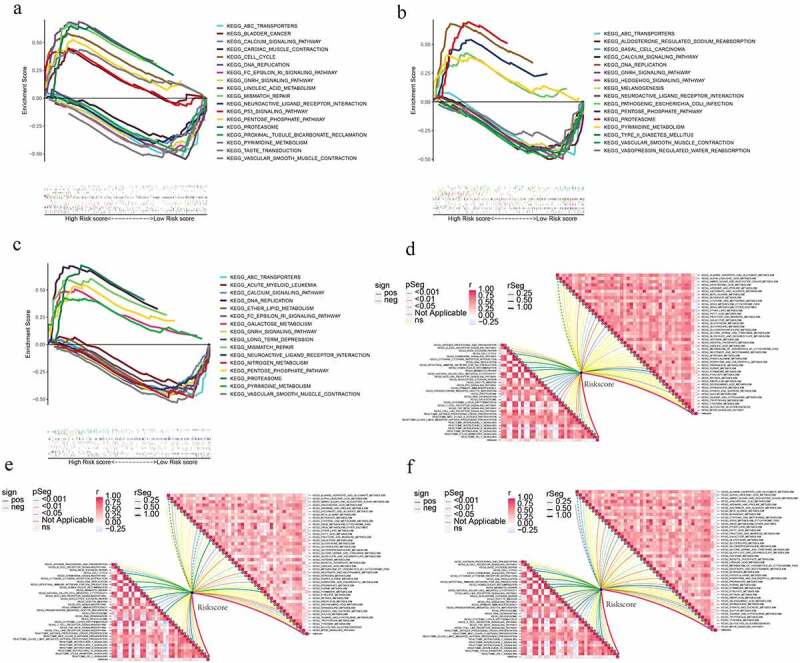
ARPS-based functional annotation. (a-c) Gene set enrichment analysis of the low- and high-risk subgroups in train, test1, and test2 cohorts. (d-f) Relationships between risk score and immune-related (in the left bottom panel) and metabolism-related pathways (in the upper right panel) in train, test1, and test2 cohorts.

As we all know, pathway dysregulation is correlated with avariety of cancers. Thus, GSEA was conducted to determine the discrepancy in pathway activities underlying the low-and high-risk subgroups, which might be responsible for the discrepancy in prognosis between the two subgroups. As illustrated in Figure 9(a–c), DNA_REPLICATION, PROTEASOME, PENTOSE_PHOSPHATE_PATHWAY, and PYRIMIDINE_METABOLISM were substantially enriched in the high-risk subgroup, while the VASCULAR_SMOOTH_MUSCLE_CONTRACTION, ABC_TRANSPORTERS, CALCIUM_SIGNALING_PATHWAY, NEUROACTIVE_LIGAND_RECEPTOR_INTERACTION, and GNRH_SIGNALING_PATHWAY were enriched in the low-risk subgroup in the training, test1, and, test2 cohorts. An examination of the correlations between risk scores and immune and metabolic illnesses was conducted due to the strong relationship between adipocytokines, immunological disorders, and metabolic disorders. For immune-related pathways, the ARPS-based risk score is negatively correlated with B_CELL_RECEPTOR_SIGNALING_PATHWAY, PRIMARY_IMMUNODEFICIENCY, and INTERLEUKIN_2_SIGNALING, but is positively correlated with P53_SIGNALING_PATHWAY, MISMATCH_REPAIR, NUCLEOTIDE_EXCISION_REPAIR, HOMOLOGOUS_RECOMBINATION, DNA_REPLICATION, CELL_CYCLE, PROTEASOME, INTERLEUKIN_1_SIGNALING, CLASS_I_MHC_MEDIATED_ANTIGEN_PROCESSING_PRESENTATION, BASE_EXCISION_REPAIR, MHC_CLASS_II_ANTIGEN_PRESENTATION, PROGESTERONE_MEDIATED_OOCYTE_MATURATION, and OOCYTE_MEIOSIS. As for metabolism-related pathways, the ARPS-based risk score had a negative correlation with ARACHIDONIC_ACID_METABOLISM, ALPHA_LINOLENIC_ACID_METABOLISM, GLYCEROPHOSPHOLIPID_METABOLISM,

FATTY_ACID_METABOLISM, ETHER_LIPID_METABOLISM, and LINOLEIC_ACID_METABOLISM while a positive correlation with PYRIMIDINE_METABOLISM, GALACTOSE_METABOLISM, GLYCEROLIPID_METABOLISM, and SULFUR_METABOLISM (Figure 9(d–f)).

### 3.8. Development and verification of nomogram plot

**Figure 10.**
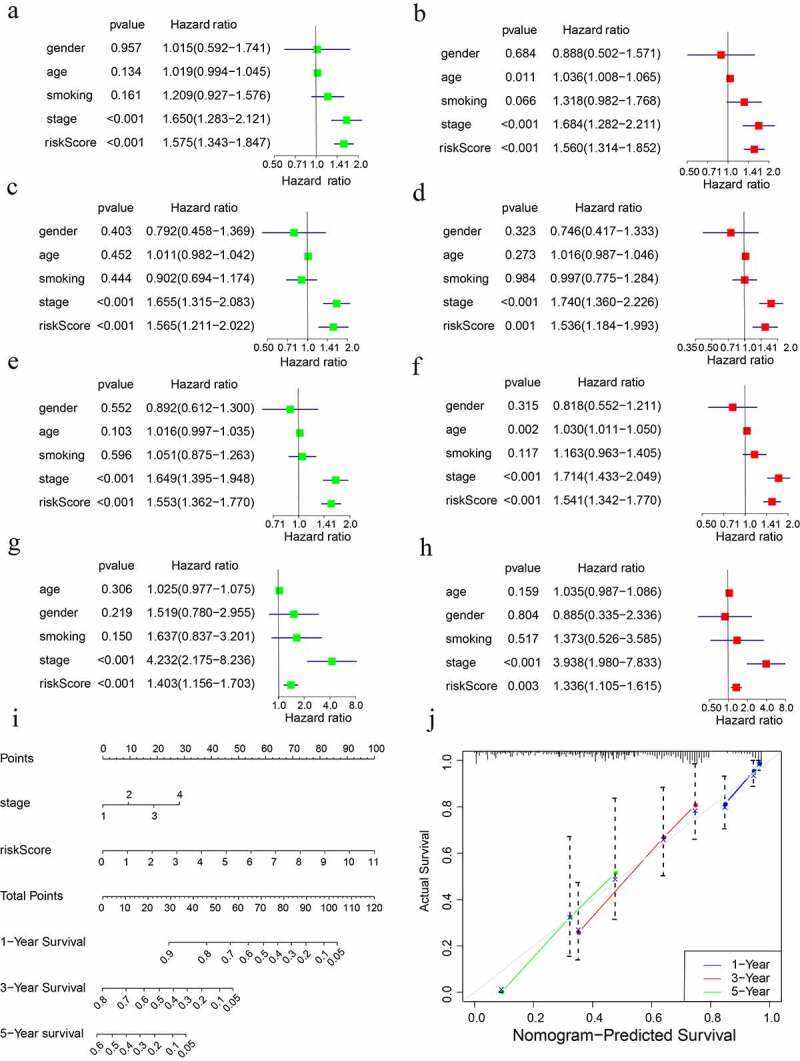
Creation and verification of the risk score-based nomogram. Multivariate and univariate cox regression analyses in (a, b) training cohort, (c, d) test1 cohort, (e, f) test2 cohort, and (g,h) test3 cohort. (i) The nomogram for survival probability over one, three, and five years. (j) Verification of the predictive power of the nomogram using calibration curves.

First, stage and risk score were identified to independently serve as prognostic indicators in the training cohort according to the findings obtained from the univariate and multivariate cox regression analyses. All the p values and hazard ratios were shown in Figure 10(a,b). Furthermore, in test1, test2, and test3 cohorts, the independent predictive function of stage and risk score in LUAD was validated (Figure 10(c–h)). Subsequently, a nomogram was generated by merging these independent prognostic variables. According to the value of each variable, the total score can be easily calculated for predicting the OS of LUAD patients over one, three, and five years (Figure 10(i)). Furthermore, calibration curves were generated in order to validate the anticipation power of the nomogram, and the findings revealed that the anticipated survival rates of the nomogram and actual survival rates were largely in agreement (Figure 10(j)).

## 4. Discussion

With the deepening of research about adipocytokines, the role of adipocytokines in cancer has been continuously explored [29–32]. Thus, we summarize the variations of adipocytokines in a variety of cancers before investigating the influence of abnormal adipocytokines in LUAD specifically. Indeed, variations of adipocytokines more or less existed in various cancers. Then, we distinguished prognostic genes from adipocytokines and adipocytokine-related lncRNAs and screened the optimized candidate genes for signature construction in order to obtain an ideal signature with clinical significance. After internal and external validation, a novel ARPS consisting of two adipocytokines and three adipocytokine-related lncRNAs (i.e. RELA, LINC01137, CAMKK1, FAM30A, and CYP1B1-AS1) was identified with satisfied prognostic performance.

The prognostic significance of these five genes in LUAD has been supported by other research studies as well. RELA, forming a positive feedback loop with AKT/MAPK, is implicated in the proliferation of LUAD [33]. Additionally, RELA can also promote lung cancer proliferation by regulating Wnt/β-catenin signalling [34]. As for CAMKK1, it is reported that the polymorphism of CAMKK1 rs7214723 was correlated with an increase in lung cancer risk [35]. What’s more, some established prognostic signatures showed the potential roles of the other three genes in LUAD. CYP1B1-AS1 is included in a redox-related prognostic signature of LUAD [36]. FAM30A is involved in an immune-related prognostic signature for LUAD [37]. LINC01137 has been reported to be included in an autophagy-related prognostic signature and a redox-related prognostic signature of LUAD [36,38].

In the training, test 1, test2, and test3 cohorts, LUAD patients can be successfully classified into the high-risk subgroup with an unfavourable prognosis and the low-risk subgroup with a relatively good prognosis with the aid of ARPS. In view of the association between adipocytokine and immune, the next section revealed the discrepancy of immune status in low- and high-risk subgroups of LUAD. As is known, cancer cells may be confused as normal constituents of the human body to afford self-protection through the immune checkpoint pathways [39]. The greater proportion of immune components but poor prognosis in the high-risk subgroup implied that immune checkpoint pathways might be active. Exactly, immune checkpoint genes are expressed differently in the two subgroups. The upregulation of LDHA, CD276, and IL23A and downregulation of PTPRC, CD48, TNFRSF14, CTLA4, JAK2, CD28, CD27, TNFRSF25, CD200R1, BTLA, CD160, CD244, and CD40LG in high-risk subgroup might become promising targets in LUAD. In subsequent in-depth exploration about the abundance of immunocyte-infiltration, although cancer-promoting Treg [40] upregulated in the low-risk subgroup, a great number of anti-tumour immune cells including B cells and most types of T cells showed up-regulation in the low-risk subgroup. In high-risk subgroups, more samples with obvious down-regulation of most anti-tumour immune cells and up-regulation of cancer-promoting Th2 existed [41,42]. The discrepancies of immune subtypes are in agreement with the results above. Characterized with a relatively lower level of cancer-promoting Th2 cells, more samples of the C3 subtype belonged to the low-risk subgroup. Besides, more samples in C1 and C2 subtypes with higher SNV neoantigens, greater intratumor heterogeneity, and stronger proliferation existed in the high-risk subtype. In addition, many immune-related pathways also differed in low- and high-risk subgroups. All these discrepancies might be responsible for different prognoses and serve as promising targets for immune therapy.

Additionally, the diagnostic value of risk score, as good as the stage, is confirmed by the time-dependent ROC curve. Notably, ARPS exhibits a stronger prediction performance for LUAD patients as opposed to other well-known signatures. Furthermore, the risk score is verified to be an independent prognostic indicator as well as the stage. LUAD patients’ survival rate was quantitatively examined after plotting a nomogram according to the risk score and stage in order to take full advantage of the prognostic potential of ARPS. Calibration curves were utilized to verify the predictive ability of the nomogram to anticipate with high accuracy.

There are a number of drawbacks of the present research that need to be taken into consideration. Firstly, the ARPS was developed using a limited number of LUAD patients from the GEO and TCGA datasets. It is thus necessary to conduct a large prospective clinical trial to confirm the prediction value of this prognostic signature. Furthermore, the ARPS was developed only via bioinformatic research, and more fundamental studies are required to validate our findings.

## 5. Conclusions

In the present research, an ARPS was effectively created and validated for the first time to accurately predict the clinical prognosis of LUAD patients. The discrepancies of ICGs, immune-related pathways, and immune response among patients with different prognoses were investigated. Subsequently, utilizing this signature and the clinical stage, a nomogram was created as a quantitative resource to support the prediction of the survival rates of LUAD patients. In conclusion, the present research may deliver new insight into clinical decision-making as well as personalized medicine for LUAD.

## Acknowledgments

We thank Bullet Edits Limited for the linguistic editing of the manuscript.

## Supplementary Material

Supplemental Material

## Data Availability

All the data are available upon request.
