# Supplementary figures and images for: Transcriptome analysis of adipocytokines and their-related LncRNAs in lung adenocarcinoma revealing the association with prognosis, immune infiltration, and metabolic characteristics
Source: Adipocyte. 2022 Apr 17;11(1):250–65. doi: 10.1080/21623945.2022.2064956 (PMC9037474; doi:10.1080/21623945.2022.2064956)

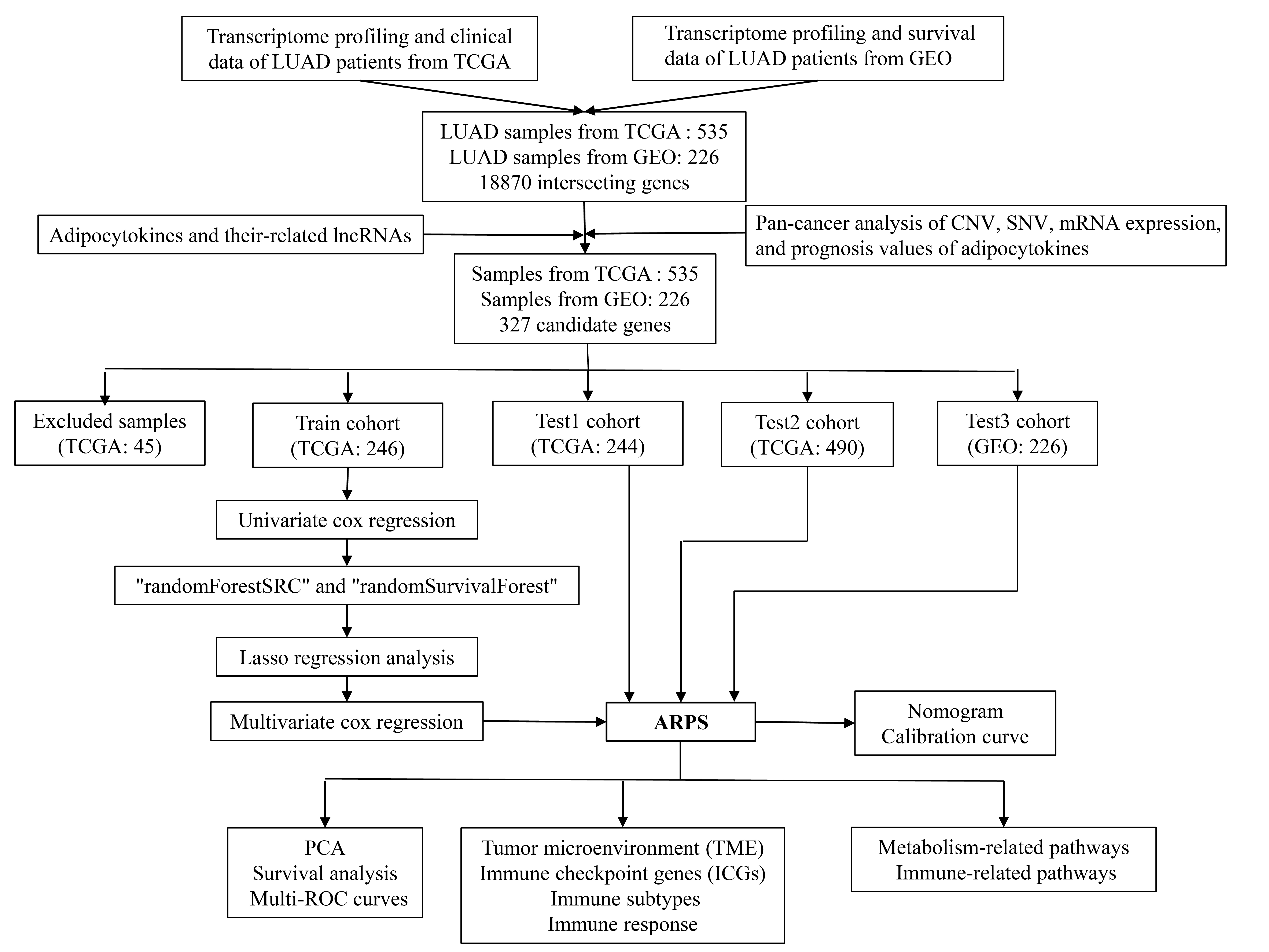

Supplement: Supplemental Material [file KADI_A_2064956_SM9978.zip › supplementary/Supplementary Figure 1.jpg]

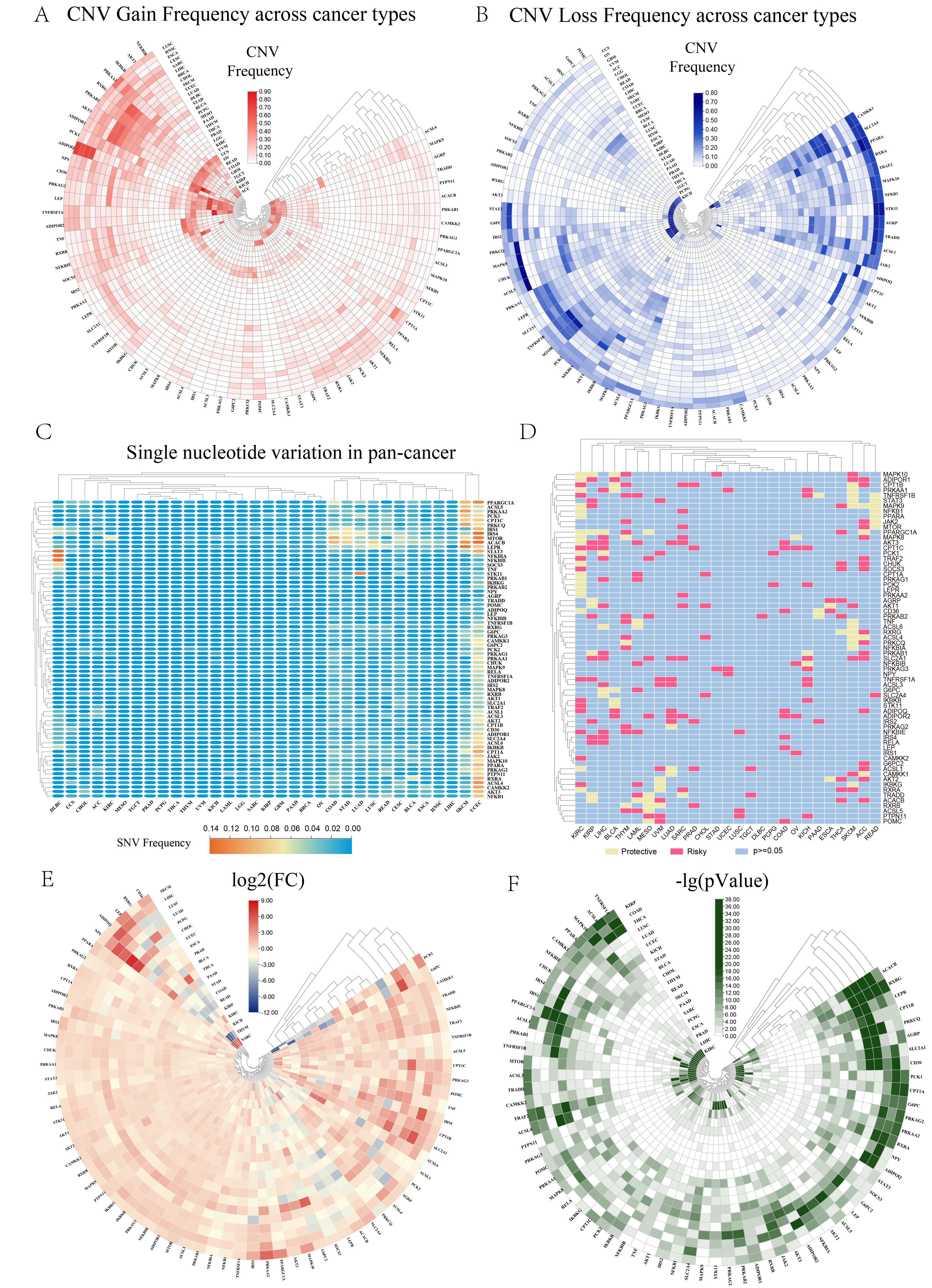

Supplement: Supplemental Material [file KADI_A_2064956_SM9978.zip › supplementary/Supplementary Figure 2.jpg]
